# Feasibility of implementing a novel behavioural smoking cessation intervention amongst human immunodeficiency virus-infected smokers in a resource-limited setting: A single-arm pilot trial

**DOI:** 10.4102/sajhivmed.v21i1.1075

**Published:** 2020-06-24

**Authors:** Billy M. Tsima, Precious Moedi, Joyce Maunge, Kitso Machangane, Martha Kgogwane, Tebogo Mudojwa, Joseph Bastian, Warren Bilker, Rebecca Ashare, Robert Schnoll, Robert Gross

**Affiliations:** 1 Department of Family Medicine and Public Health, Faculty of Medicine, University of Botswana, Gaborone, Botswana; 2 Princess Marina Hospital, Dental Department, Gaborone, Botswana; 3 Botswana UPenn Partnership, Gaborone, Botswana; 4 Department of Psychiatry, Perelman School of Medicine, University of Pennsylvania, Philadelphia, United States of America; 5 Department of Biostatistics, Epidemiology and Informatics, Perelman School of Medicine, University of Pennsylvania, Philadelphia, United States of America; 6 Department of Medicine (ID), Perelman School of Medicine, University of Pennsylvania, Philadelphia, United States of America

**Keywords:** smoking cessation, tobacco, behaviour activation, problem solving, HIV

## Abstract

**Background:**

Tobacco use is prevalent amongst individuals infected with human immunodeficiency virus (HIV). In resource-constrained settings, pharmacological smoking cessation interventions are unfeasible because of their high cost. There is a need to develop and evaluate behavioural interventions to address the unique challenges of tobacco use in the HIV-infected populations in these settings.

**Objectives:**

The authors aimed to assess the feasibility and acceptability of the Behavioural Activation/Problem Solving for Smoking Cessation (BAPS-SC) intervention programme to determine whether it should be tested in an adequately powered randomised controlled trial.

**Method:**

The authors merged behavioural activation therapy (BAT) with the principles of problem-solving therapy to create a novel five-session counselling model to address the unique challenges of tobacco cessation amongst those infected with HIV. Feasibility measures included the rate of enrolment amongst those eligible and the retention rate and descriptive analysis of intervention acceptability. The authors’ secondary outcome was 7-day point smoking prevalence abstinence, confirmed with breath carbon monoxide.

**Results:**

A total of 128 individuals were screened over 8 weeks with 50 deemed eligible and 40 enrolled (80%). Retention at week 12 was 53% (21/40). The 7-day point prevalence abstinence, co-confirmed, at week 12 was 37.5% (15/40). All respondents indicated that they would recommend BAPS-SC to other smokers who want to quit, and would be willing to participate in the programme again up to the point of exit if they did not stop smoking.

**Conclusion:**

A full-scale randomised control trial comparing BAPS-SC with usual practice is warranted to evaluate the efficacy of this novel intervention in these settings.

## Introduction

The human immunodeficiency virus (HIV) epidemic in sub-Saharan Africa has resulted in a large-scale transformation of healthcare delivery in heavily affected countries such as Botswana.^1^ Unfortunately, other health threats such as cardiovascular disease and cancer have emerged amongst people living with HIV/AIDS (PLWHA) partly because of the chronic inflammation of HIV that is compounded by high rates of smoking in this population.^2^ As such, addressing modifiable cardiovascular risk factors amongst those with HIV infection, including tobacco use, has become a critical priority.^3,4^

Indeed, continued smoking amongst those with HIV infection can result in serious adverse effects, including reduced effectiveness of antiretroviral (ARV) therapy.^5^ Controlling for medication adherence and comorbid illicit drug use, HIV-infected smokers on ARV have a significantly lower likelihood of achieving a viral response and a greater chance of viral or immunologic failure compared with their non-smoking counterparts.^6^ Persistent smoking amongst HIV-infected individuals may also increase progression to acquired immunodeficiency syndrome (AIDS).^7^ Pulmonary tuberculosis, a major public health crisis in sub-Saharan Africa, occurs at higher rates in smokers compared with non-smokers.^8^ Overall, the mortality gains of ARV treatment and the associated improved quality of life amongst those with HIV infection are being jeopardised by the cardiovascular and neoplastic diseases attributable to tobacco use in this population.^9^

Although interest in quitting smoking is high amongst PLWHA,^10,11,12^ particularly when HIV treatment is initiated^13^ and when tobacco use treatment is integrated with HIV care,^14^ remarkably little research has focussed on developing and testing smoking cessation interventions for PLWHA.^11^ Unfortunately, in low-income and developing countries, the costs of pharmacotherapy for nicotine addiction make pharmacological treatments currently inaccessible for PLWHA and further emphasise the importance of novel behavioural strategies.

Depressive symptoms are common in HIV-infected populations, often comorbid with smoking, and associated with poor smoking cessation rates.^15^ Behavioural activation therapy (BAT), rooted in a behavioural economic framework, has been effective at treating depression, and preliminary data in the United States of America (USA) suggests that it may also effectively address smoking.^16^ Behavioural activation therapy aims to increase engagement in healthy rewarding activities (i.e., alternative reinforcers) by reducing patterns of avoidance, withdrawal and inactivity, and to decrease activities that enhance the rewarding aspects of smoking (i.e., complementary reinforcers). Additionally, problem-solving approaches have been used with PLWHA to improve medication adherence and decrease depressive symptoms.^17^ Behavioural activation therapy and problem-solving approach may help smokers select and implement activities that will replace smoking, thereby reducing smoking rates.

The authors developed a novel counselling model incorporating elements of behavioural activation and problem solving to address the unique challenges of tobacco cessation amongst those with HIV infection. They aimed to assess the feasibility and appeal of Behavioural Activation/Problem Solving for Smoking Cessation (BAPS-SC) intervention to determine whether it should be tested in an adequately powered clinical trial.

## Methods

### Participant enrolment

The authors conducted a single-arm pilot trial of the BAPS-SC intervention in Botswana amongst HIV-infected individuals aged 18–65 years who smoked ≥ 5 cigarettes/day, on average, at four outpatient HIV clinics. The target accrual goal was 40 participants. This was based on the assumption that at least five participants would be enrolled per week, thus ensuring that the recruitment of human subjects into the trial is timely as this is vital to the success of the trial.^18^ The authors excluded participants if they reported current untreated and unstable alcohol dependence; current use or discontinuation within last 14 days of smoking cessation medications; current diagnosis of unstable and untreated major depression or current or past diagnosis of psychotic disorder; use of chewing tobacco, snuff or snus; current participation in a smoking cessation programme; or plans to use nicotine substitutes or smoking cessation treatments in the next 7 months. The restriction to 7 months was based on the anticipated period to conduct the trial so as to avoid contamination of the intervention with other smoking cessation modalities not under investigation.

A trained recruiter approached each patient in clinic to ascertain smoking status. Those acknowledging smoking were asked to participate in a questionnaire related to their smoking and offered participation in the pilot trial. Those who agreed were referred to the research assistant who arranged to meet with them to determine eligibility and administer informed consent.

### Design of intervention

The authors merged BAT with the principles of problem-solving therapy to create a novel five-session counselling model to address the unique challenges of tobacco cessation amongst PLWHA (BAPS-SC). Members of the team created a formal treatment manual, which was evaluated for cultural appropriateness by Botswana co-investigators, including assessment of translation and back translation and pre-pilot testing using videoconferencing to role play. Key components of BAPS-SC include activity monitoring and rewarding activity scheduling, assessment of personal goals and values, assessment and altering of avoidance behaviour and other maladaptive coping strategies, and contingency management. Behavioural Activation/Problem Solving for Smoking Cessation focuses on reducing stress pile-up and loss of pleasure that accompanies the cessation process and on identifying and establishing environmental/social changes to promote abstinence. Behavioural Activation/Problem Solving for Smoking Cessation addresses smoking as a behaviour that prevents and restricts opportunities for contact with healthy rewarding behaviours. These changes are achieved through altering daily routines previously associated with smoking in ways that increase pleasure and mastery across life domains, reducing rumination and increasing behavioural skills to prevent return to smoking as a means of avoiding stressors.

A pre-quit session (session 1) introduces participants to: (1) self-monitoring of mood and behaviour; (2) assessment of personal values to refine the treatment plan; and (3) scheduling of substitute rewarding activities that align with their abstinence goal. At the target quit date (TQD) session (session 2), participants’ experiences with abstinence are reviewed and functional analysis of behaviour is introduced, especially as it relates to smoking and avoidance patterns. Information obtained is used to help generate a tailored behavioural activation plan by using the problem-solving framework to increase rewarding activities and relationships, reduce avoidant responses to distressing experiences and facilitate successful implementation of smoking trigger management strategies. Sessions 3–5 incorporate strategies to address avoidance patterns, especially those involving smoking, and replace them with adaptive coping strategies, again by using problem solving. Sessions were conducted by telephone over a 12-week period (with the first session lasting 1 h and subsequent sessions lasting 30–45 min on average) and involved weekly homework assignments.

### Data collection and management

Collected data included information related to smoking behaviour (including nicotine dependence measured by the Fagerström Test for Nicotine Dependence^19^), anhedonia [using the Snaith–Hamilton Pleasure Scale (SHAPS)^20^] and feasibility (e.g., rate of accrual and retention and appeal of the intervention). Participant accrual rate was defined as the number of participants enrolled over 8 weeks. Retention was calculated as a proportion of participants enrolled who were available at week 12 contact session. Study staff not acting as interventionists interviewed participants at baseline, week 6 and week 12 to determine if they were still smoking and whether they implemented the suggested intervention strategies. The timeline follow-back procedure assessed daily smoking between measurement time points.^21^ Additionally, the amount of carbon monoxide (CO) in participants’ breath was tested to confirm self-reported abstinence from tobacco. Data were collected on paper and transferred to an electronic REDCap database for analysis.

### Data analysis

The authors used descriptive statistics to characterise the sample. Feasibility measures included the rate of enrolment and retention and descriptive analysis of intervention acceptability (e.g., whether the participants would have enrolled in the intervention had they known what the experience would be like and whether they would refer a friend who wanted to quit smoking). The primary efficacy outcome was 7-day point prevalence abstinence at week 12 (12 weeks post-TQD), defined as self-reported abstinence for 7 days prior to the assessment and breath CO <8 ppm.^22^ This approach is in line with the recommendations of the working group of the Society for Research on Nicotine and Tobacco based on literature review of abstinence measures used in trials of smoking cessation intervention.^23^

### Ethical consideration

Ethical approval was obtained from the Botswana Ministry of Health (Health Research Unit, reference number: HPDME 13/18/1).

### Results

**FIGURE 1:**
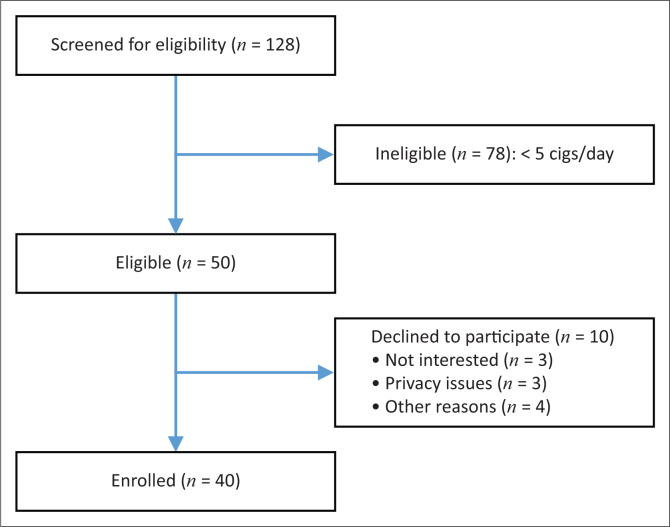
Participant flow diagram.

Characteristics of the sample are described in Table 1. A total of 128 individuals were screened over 8 weeks with 50 deemed eligible and 40 enrolled (80%), as shown in Figure 1. Retention at week 12 was 52.5% (21/40).

**TABLE 1:** Demographic characteristics.

Characteristic	*N* or Median	% or IQR
Age in years	39.5	34, 48
Male candidates	38	95
Number of cigarettes per day	10	6, 11.5
FTND category at enrolment (week 0)	-	-
Low dependence (1–2)	0	-
Low-to-moderate dependence (3–4)	0	-
Moderate dependence (5–7)	37	92.5
High dependence (≥8)	3	7.5
SHAPS score at enrolment	1.5	0.5, 2.5
SHAPS score > 2	10	25

IQR, interquartile range; FTND, Fagerstr öm Test for Nicotine Dependence; SHAPS, Snaith–Hamilton Pleasure Scale (normal = 2 or less; abnormal = 3 or more).

The 7-day point prevalence abstinence at week 12 was 37.5% (15/40). Notably, all respondents indicated that they would recommend BAPS-SC to other smokers who want to quit and would be willing to participate in the programme again up to the point of exit if they did not stop smoking. When a more stringent threshold of exhaled CO ≤ 4 ppm was used as the criterion for success, the quit rate in this pilot was 9/40 (22.5%).

## Discussion

The authors conducted a single-arm pilot trial of the BAPS-SC intervention programme, a novel behavioural smoking cessation intervention for PLWHA in a high-HIV-burden sub-Saharan country wherein all 40 participants received the intervention. This design is ideal for studies aiming to determine if a novel intervention is appealing and feasible in the setting and warrants a full-scale clinical trial.^24^ The results of the CO monitoring and exit interviews suggest that BAPS-SC is likely to be efficacious.

This study’s results of a high degree of smoking cessation success with BAPS-SC suggest a potentially highly impactful smoking cessation programme given that behavioural smoking cessation programmes rarely yield quit rates higher than 10% – 15%.^25^ There is no comparable trial that tested the intervention developed by the team of the authors with an HIV-negative cohort of smokers. Two comparisons are worth noting, and both indicate a substantial benefit from authors’ intervention. Firstly, meta-analyses of behavioural interventions (without medications) for smoking cessation rooted in classic cognitive-behavioural theory show that they yield quit rates of generally less than 15% at the end of treatment.^26^ Secondly, MacPherson et al. (2010), in their pilot test of BAT for smoking cessation, reported an end-of-treatment quit rate of 17%.^27^ The authors used a higher threshold of CO to determine smoking cessation as opposed to a more stringent cut-off in the range of 3 ppm – 4 ppm recently proposed by other researchers.^28^ However, 8 ppm is the cut-off used in most smoking cessation clinical trials for PLWHA, so the authors chose this cut-off to compare their results to the current literature. With regard to past behavioural smoking cessation treatments for PLWHA, a cell phone-based intervention was found to be associated with significantly higher initial quit rates compared with usual care.^29^ Other studies including a group-based tailored intervention, motivational interventions and a web-based intervention (vs in-person or self-help) have not yielded significant increases in quit rates.^30,31,32,33,34,35,36^ Two pilot studies of behavioural treatments that address negative affect (depression and anxiety) show promise for PLWHA.^37,38^

The BAPS-SC trial proved feasible. Firstly, the target sample size of 40 participants was reached within 8 weeks of recruitment, a rate of enrolment that would make large trials feasible. The authors’ findings indicated that a large-scale clinical trial would be feasible to determine the efficacy of the BAPS-SC programme in this setting where HIV prevalence is relatively high. Furthermore, smoking prevalence in the setting of their pilot study was estimated to be as high as 51% for male candidates and 6% for female candidates with HIV infection.^39^ There were proportionately fewer female candidates enrolled in the present study consistent with data from demographic and health surveys in sub-Saharan Africa.^40^ Although these smoking rates appear to be comparatively lower than reports from North America and Europe, these rates are projected to increase in the African continent whilst they are falling in other parts of the world.^40,41^ Given the limited behavioural health infrastructure in low middle income countries (LMICs) such as Botswana, the authors leveraged HIV clinical care sites and telephone-delivered counselling to extend the reach of skilled practitioners. The authors found this strategy to be effective and acceptable by the participants in the pilot trial.

Smoking is a major cause of morbidity and mortality, yet smokers find it very difficult to quit. If individuals quit because of this intervention, they will reduce their risk of cardiovascular disease, chronic obstructive pulmonary disease and cancers, particularly lung cancer. This will be of direct benefit to the individual participant. Further, if the authors are able to mount a full-scale trial based on the results of this pilot trial, high HIV burden LMICs such as Botswana may benefit because the intervention may become standard of care in the country, reducing disease burden caused by tobacco use. Being a behavioural intervention without pharmacological intervention, the cost is expected to be substantially less with the BAPS-SC compared with a pharmacological intervention. A formal cost-effective analysis will be needed to be undertaken following the roll out of the programme.

Results of the exit questionnaires to evaluate the acceptability of the BAPS-SC trial indicate that HIV-infected smokers in Botswana find the programme to be appealing and acceptable. The results indicate a lower-than-expected retention rate with slightly over half of the participants remaining in the study after a 12-week follow-up period. The retention rates across most smoking cessation trials generally exceed 75%.^42^ This may be improved by asking participants to provide contact numbers of associates such as friends and family members who can be contacted if the participant is unreachable after a number of attempts and increasing incentives to complete follow-ups. From an evidence-based medicine perspective, if efficacy of the intervention is determined to be high, implementation science methods would need to be employed in the clinical care setting to incorporate the intervention into HIV programmes. The intervention leverages the existing HIV care infrastructure and will likely facilitate scale-up in sub-Saharan African settings where HIV is common and smoking continues to emerge as a threat to HIV-positive individuals’ health and survival.

### Limitations

This pilot study evaluating the feasibility of BAPS-SC amongst HIV-infected smokers in Botswana has notable limitations. This was a single-arm trial, and therefore the results indicate only preliminary evidence of efficiency of the intervention and do not confirm efficacy. However, the authors’ main aim was to evaluate feasibility of implementing the intervention. The planned full clinical trial informed by these results will be powered to address efficacy. Additionally, the follow-up period was of only 12 weeks as the aim was to evaluate feasibility of the novel intervention. Thus, the follow-up period was not long enough to assess long-term abstinence. However, this was a pilot study to inform a future clinical trial.

## Conclusion

The results of this single-arm pilot trial demonstrate the feasibility of leveraging HIV clinical infrastructure for implementing BAPS-SC as a smoking cessation intervention programme amongst HIV-infected smokers in a resource-limited setting with high HIV burden. A full-scale clinical trial comparing BAPS-SC with standard counselling is thus warranted to evaluate the efficacy of this novel intervention in these settings.
